# Supplementary material for: Machine Learning-Based Beam Delivery Time Model for Mevion S250i With Hyperscan Technology
Source: Int J Part Ther. 2026 Feb 2;19:101301. doi: 10.1016/j.ijpt.2026.101301 (PMC12924178; doi:10.1016/j.ijpt.2026.101301)
Supplement: Supplementary file 1 — Supplementary material [file mmc1.docx]

# Supplementary Materials

## Random forest BDT model

Figure [S1](#fig:flowchart) summarizes the methodology developed to build the AI-based BDT model. At first, the treatment log files of 11 patients were collected, and then, through feature engineering, only a limited number of variables were extracted to train the model. Model training was performed on 70% of the full database combined with 5-fold cross-validation. The model performance was tested on the remaining 30% of the dataset. In addition, two clinical applications, volumetric repainting and 4D dose calculations, were analyzed to assess the model’s performance in clinical settings.


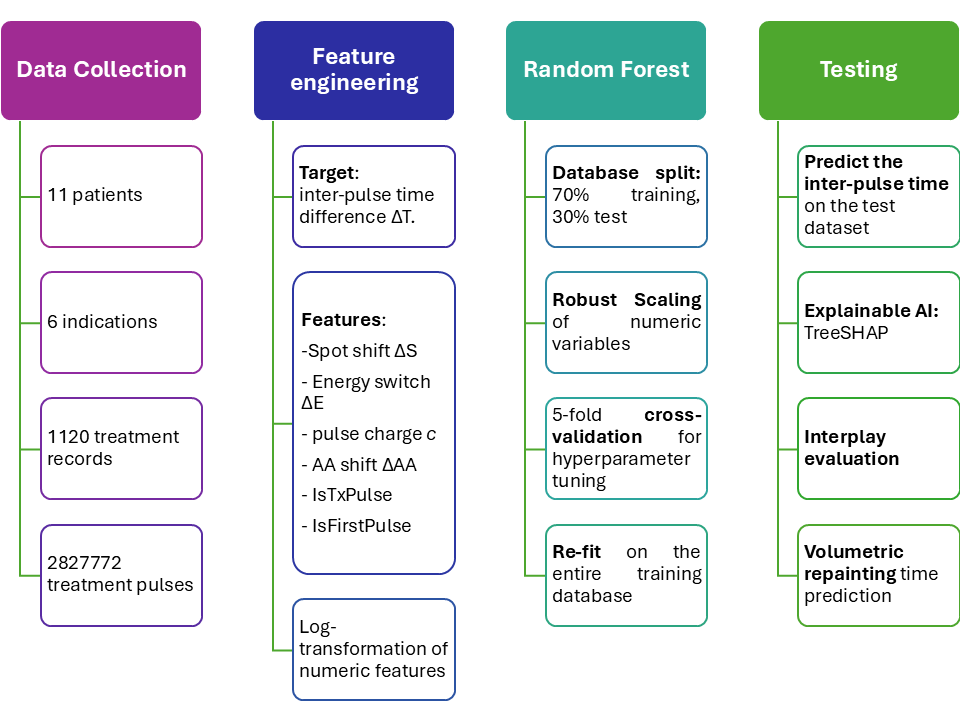


Figure S1. Flowchart the workflow for the AI-based model training and testing begins with collecting treatment records. Feature engineering is then performed to extract the target variable and model features; the model is trained and tuned, and finally, the model’s performance is evaluated on the independent dataset.

Table [S1](#tab:features) summarizes the names, type and description of the features used as input for training the RF model.

Table S1. The table summarizes the names, types, and descriptions of the features used to train the RF model. n = Numeric b = Boolean

| **Feature** | **Type** | **Description** |
| --- | --- | --- |
| $\Delta AA$ (mm) | n | AA position difference between pulses |
| $\Delta S$ (mm) | n | Spot position difference between pulses |
| $\Delta E$ (MeV) | n | Pulse energy in mega-electronvolts |
| $\Delta c$ (pC) | n | Target pulse charge difference |
| $c$ (pC) | n | Original target pulse charge |
| IsFirstPulse | b | Indicates if the pulse is the first in a sequence |
| IsTxPulse | b | Indicates if the pulse is a transmission pulse |

Table [S2](#tab:rf_params) shows the best values of the RF hyperparameters selected from the five-fold cross-validation process.

Table S2. Random Forest hyperparameter values after 5-fold cross-validation tuning.

| **Parameter** | **Value** |
| --- | --- |
| bootstrap | True |
| max_depth | 28 |
| max_features | 0.7 |
| max_samples | 0.89 |
| min_samples_leaf | 2 |
| min_samples_split | 5 |
| n_estimators | 188 |

## RF robustness towards feature redundancy and correlation

Developed four different models to test the RF robustness towards feature redundancy and correlation. Starting from the base features, we derived the following additional variables. *Log-Transformed Features:* To mitigate skewness in the distributions of key continuous variables, logarithmic transformations were applied:

- log_{\Delta AA} = $\log\left( 1+\Delta AA \right)$
- log_{\Delta S} = $\log\left( 1+\Delta S \right)$
- log_{\Delta E} = $\log\left( 1+\left| \Delta E \right| \right)$

*Interaction Features:* Interaction terms were introduced to capture compound effects:

- AA_Spot_Interaction = \Delta AA $\times$ \Delta S
- Energy_AA_Interaction = $\left| \Delta E \right|\times\Delta AA$
- Energy_Spot_Interaction = $\left| \Delta E \right|\times\Delta S$

*Categorical Binning* To model non-linear relationships, continuous variables were discretized into categorical bins:

- Energy_Category: Binned into Zero (0-0.1 MeV), Low (0.1-50 MeV), Medium (50-100 MeV), High (100-150 MeV), and VeryHigh ($>$150 MeV).
- AA_Category: Binned into Zero (0-0.1 mm), Small (0.1-10) mm, Medium (10-100 mm), and Large ($>$ 100 mm).

*Boolean Indicators:* Binary features were created to flag specific conditions:

- Is_Energy_Change: Indicates energy variation ($>0$ MeV)
- Is_Major_AA_Change: Indicates substantial aperture adjustment ($>2$ mm)
- Is_Major_Spot_Change: Indicates notable spot position change ($>1$ mm)

*Movement Metrics:* A composite movement indicator was defined to quantify overall physical change:

$$\begin{matrix} \text{Total\_Movement} & =\sqrt{{\Delta AA}^{2}+{\Delta S}^{2}+\left| \Delta E \right|^{2}} \\ \text{log\_Total\_Movement} & =log\left( 1+\text{Total\_Movement} \right) \end{matrix}$$

We developed four models with different features to test the impact of redundant or correlated features:

- **Model A**: All features without log-transformed
- **Model B**: All features without raw features (e.g. $\Delta AA$,$\Delta S$, $\Delta c$, Total_Movement)
- **Model C:** All features without interaction Features
- **Model D:** All features (Base, log-transformed, interaction, categorical, boolean)

For each model, we repeated the model training, 5-fold cross-validation, and testing to assess the model performance with the additional variables. Figures [S2](#fig:modelA), [S3](#fig:modelB), [S4](#fig:modelC), [S5](#fig:modelD) show the different models’ predictions on the test dataset. Negligible variations were observed among the different models, proving the robustness of the RF ensembles towards variable redundancy.


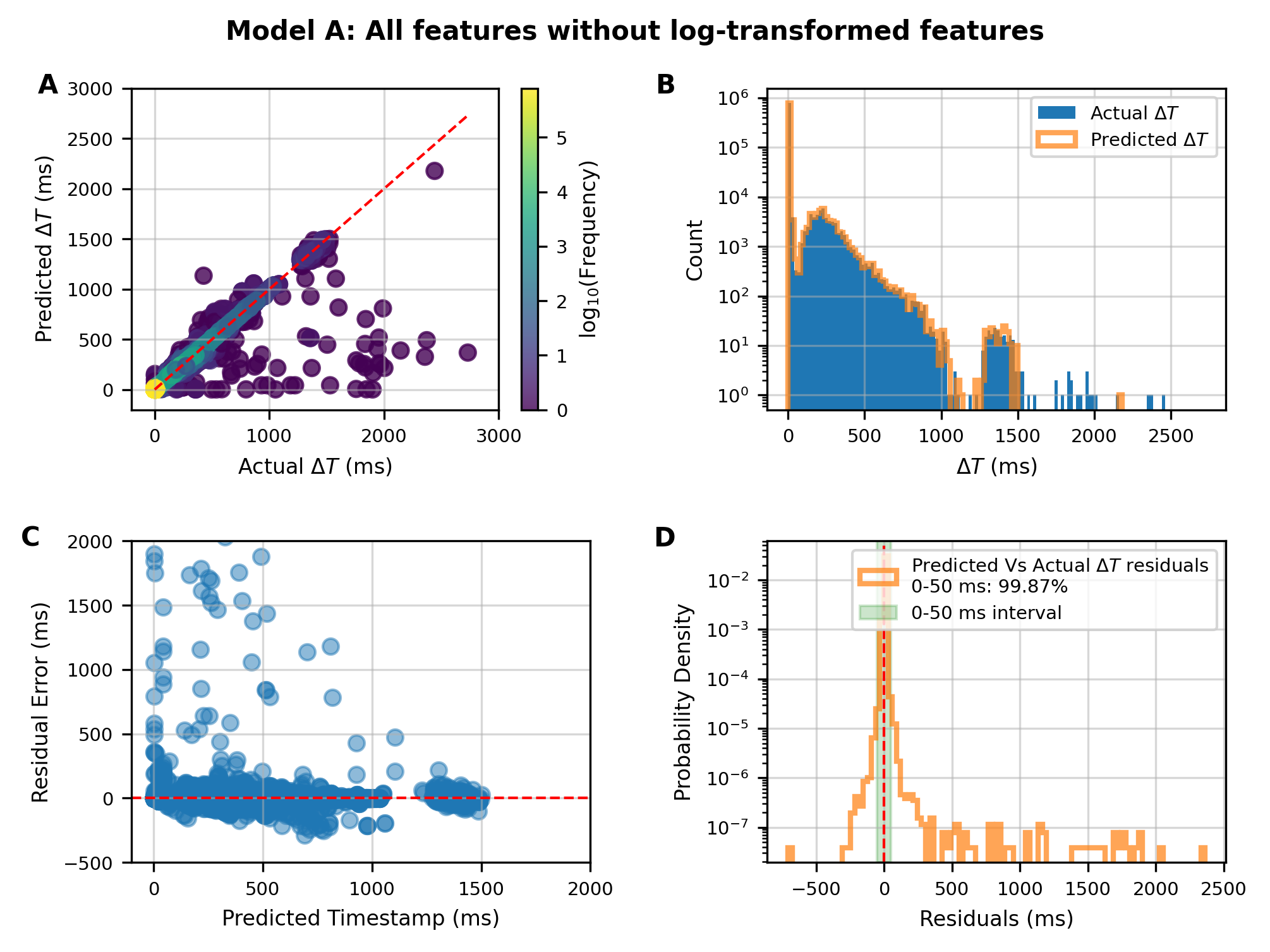


Figure S2. Evaluation of model performance and residual distribution. (A) Predicted $\Delta T$ (ms) versus actual $\Delta T$ (ms), colored by event density. (B) Histogram of event counts across $\Delta T$ intervals for actual and predicted values. (C) Residual error (ms) versus predicted timestamp (ms). (D) Probability density of residuals with highlighted 0-50 ms interval.


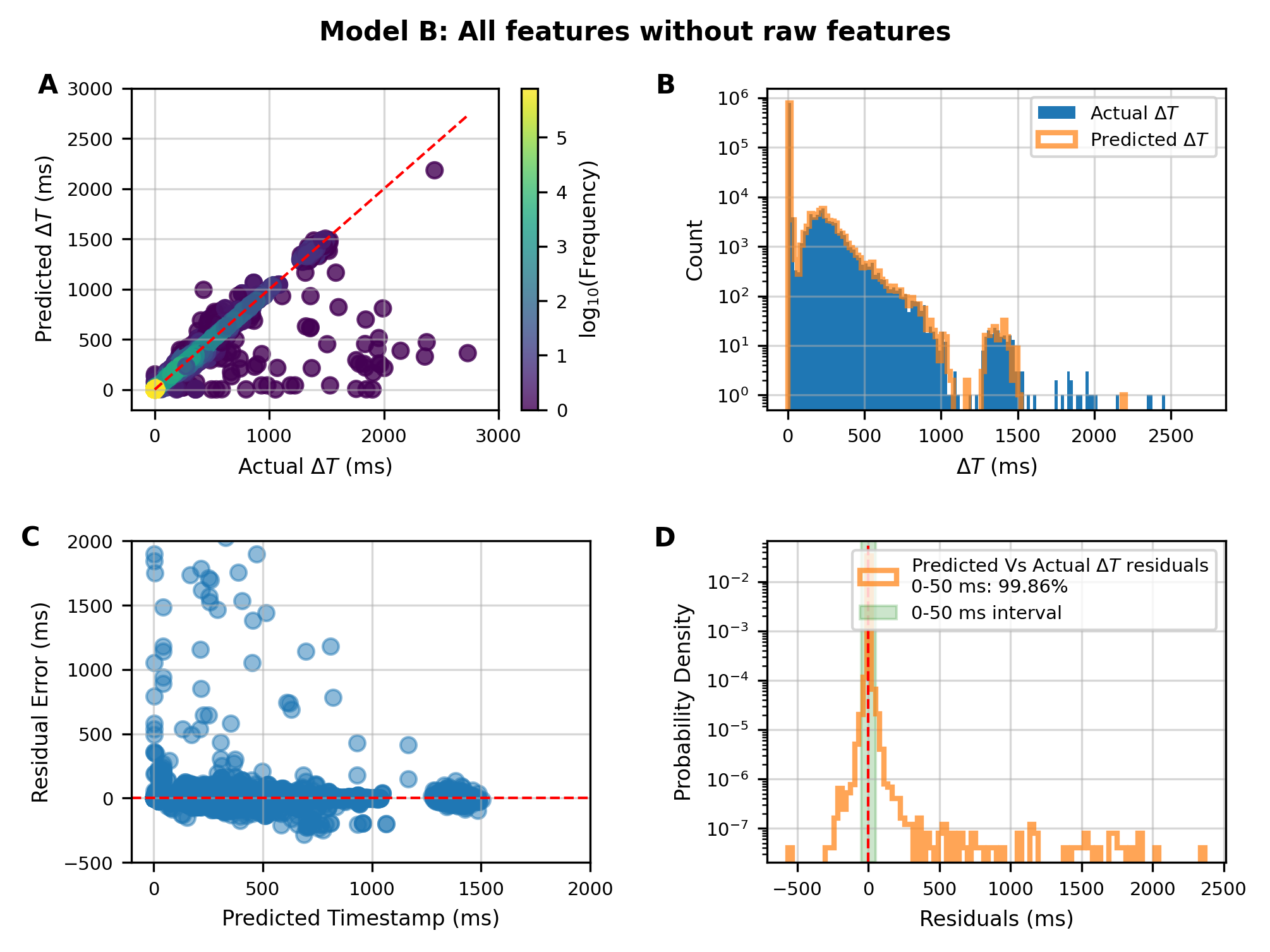


Figure S3. Evaluation of model performance and residual distribution. (A) Predicted $\Delta T$ (ms) versus actual $\Delta T$ (ms), colored by event density. (B) Histogram of event counts across $\Delta T$ intervals for actual and predicted values. (C) Residual error (ms) versus predicted timestamp (ms). (D) Probability density of residuals with highlighted 0-50 ms interval.


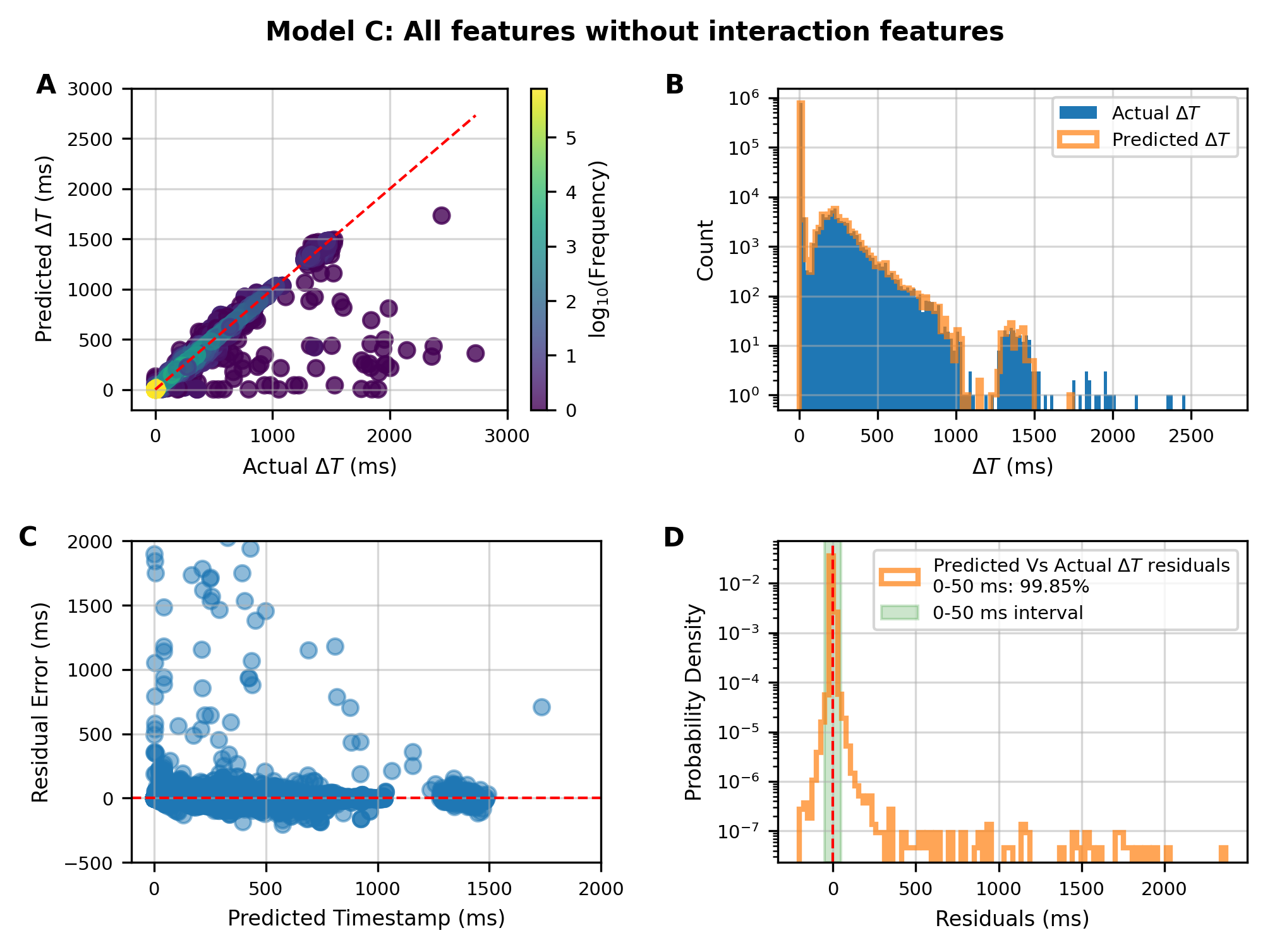


Figure S4. Evaluation of model performance and residual distribution. (A) Predicted $\Delta T$ (ms) versus actual $\Delta T$ (ms), colored by event density. (B) Histogram of event counts across $\Delta T$ intervals for actual and predicted values. (C) Residual error (ms) versus predicted timestamp (ms). (D) Probability density of residuals with highlighted 0-50 ms interval.


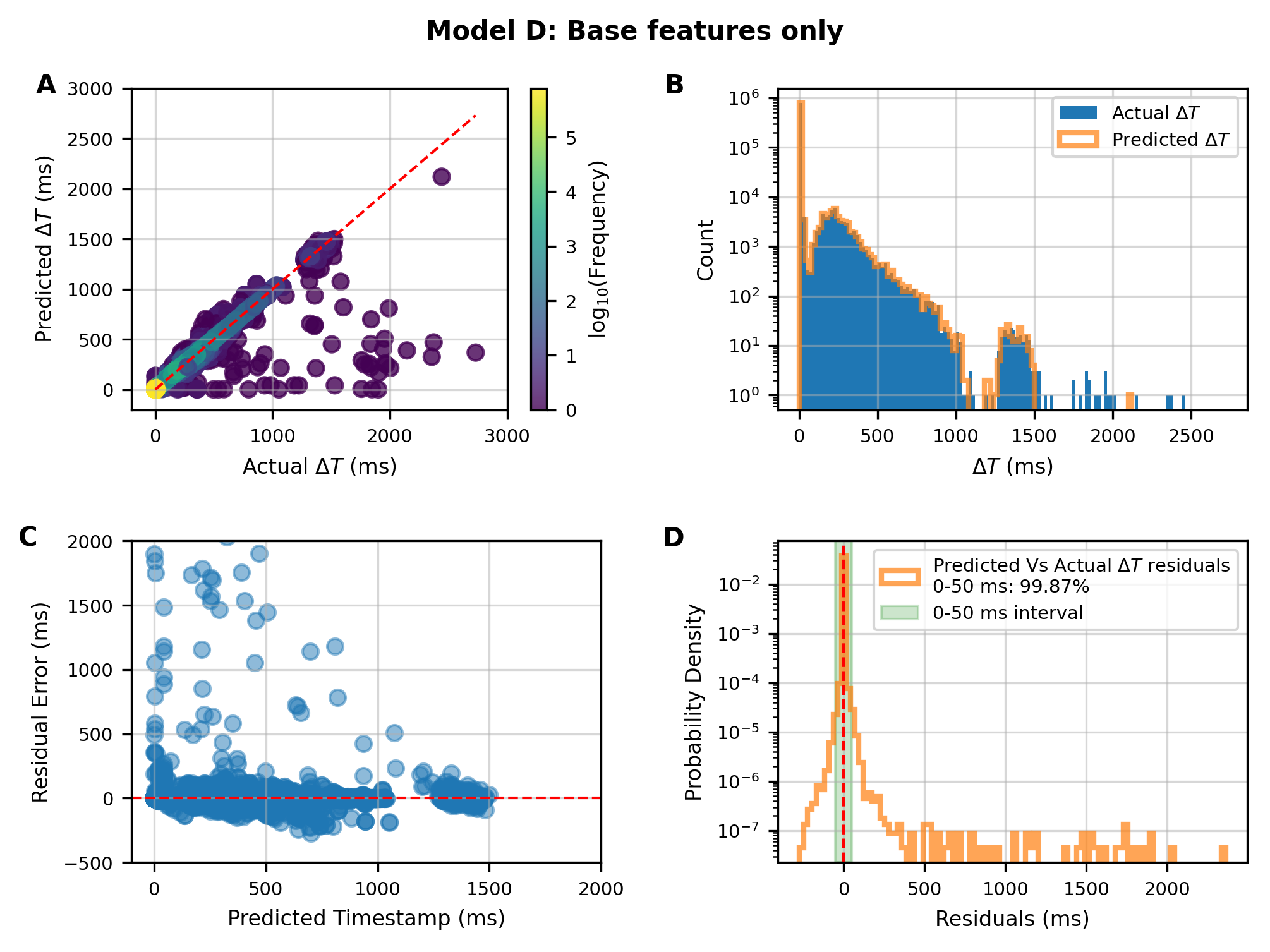


Figure S5. Evaluation of model performance and residual distribution. (A) Predicted $\Delta T$ (ms) versus actual $\Delta T$ (ms), colored by event density. (B) Histogram of event counts across $\Delta T$ intervals for actual and predicted values. (C) Residual error (ms) versus predicted timestamp (ms). (D) Probability density of residuals with highlighted 0-50 ms interval.

Table S3. MAE and MAPE values at different $\Delta T$ ranges for the different models. In brackets were reported the standard deviations of the absolute and percentage error. Model A uses all features without log-transforming, Model B uses all features without raw features, Model C uses all features without interaction features, and Model D uses all features.

| **Time range** | **Model A** | | **Model B** | | **Model C** | | **Model D** | |
| --- | --- | --- | --- | --- | --- | --- | --- | --- |
|  | **MAE [ms]** | **MAPE [%]** | **MAE [ms]** | **MAPE [%]** | **MAE [ms]** | **MAPE [%]** | **MAE [ms]** | **MAPE [%]** |
| **0–50 ms** | 0.9 (0.9) | 23.8 (21.8) | 0.9 (0.9) | 23.7 (21.7) | 0.9 (0.9) | 23.6 (21.6) | 0.9 (0.9) | 36.0 (37.2) |
| **50–500 ms** | 5.5 (13.2) | 2.2 (5.0) | 5.5 (13.1) | 2.3 (5.1) | 6.2 (13.3) | 2.5 (5.3) | 6.1 (13.3) | 2.5 (5.3) |
| **500–1000 ms** | 9.1 (42.7) | 1.4 (5.9) | 9.2 (42.8) | 1.4 (5.9) | 9.2 (42.5) | 1.7 (5.8) | 10.7 (42.4) | 1.7 (5.8) |
| $>$**1000 ms** | 209.8 (488.2) | 12.4 (26.9) | 206.6 (487.7) | 12.1 (26.8) | 220.0 (492.2) | 13.1 (27.2) | 222.0 (491.8) | 13.2 (27.1) |

## Explainable AI: Energy feature

SHAP value of $log_{\Delta E}$ feature as a function of the predicted $\Delta T$ (Figure [S6](#fig:shapEnergy)). The color map highlights the feature value.


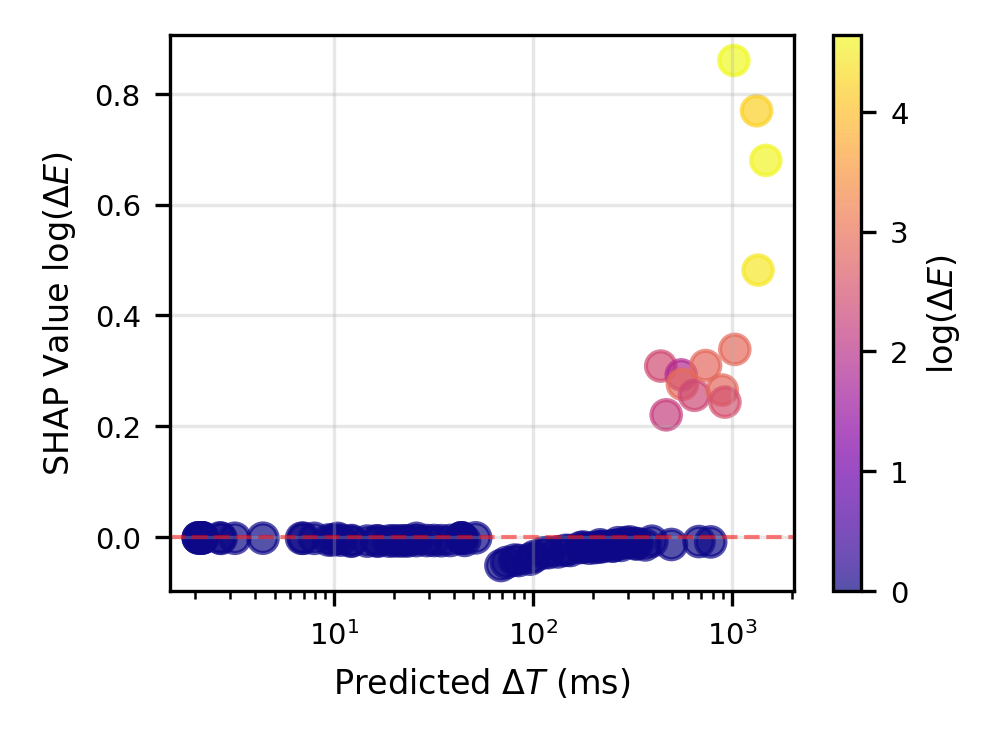


Figure S6. SHAP value for log($\Delta$E) versus predicted $\Delta T$ (ms), colored by log($\Delta E$).

## BDT uncertainty on cumulative time

To assess the uncertainty cumulated by the model for every pulse, we compared the predicted cumulative time to the actual values from the treatment log file for the repainting plan. The plan was delivered with 5 volumetric repainting, which prolonged the treatment delivery. This is a perfect case to assess the cumulative error of our model over a long treatment delivery. Figure [S7](#fig:CumulativeErrorRepainting) shows the cumulative $\Delta T$ as a function of the pulse index for the three delivered beam angles. The relative error between the model prediction and the actual values highlights a systematic underestimation of the delivery time from the RF model of on average -1.6% at the end of each beam delivery.


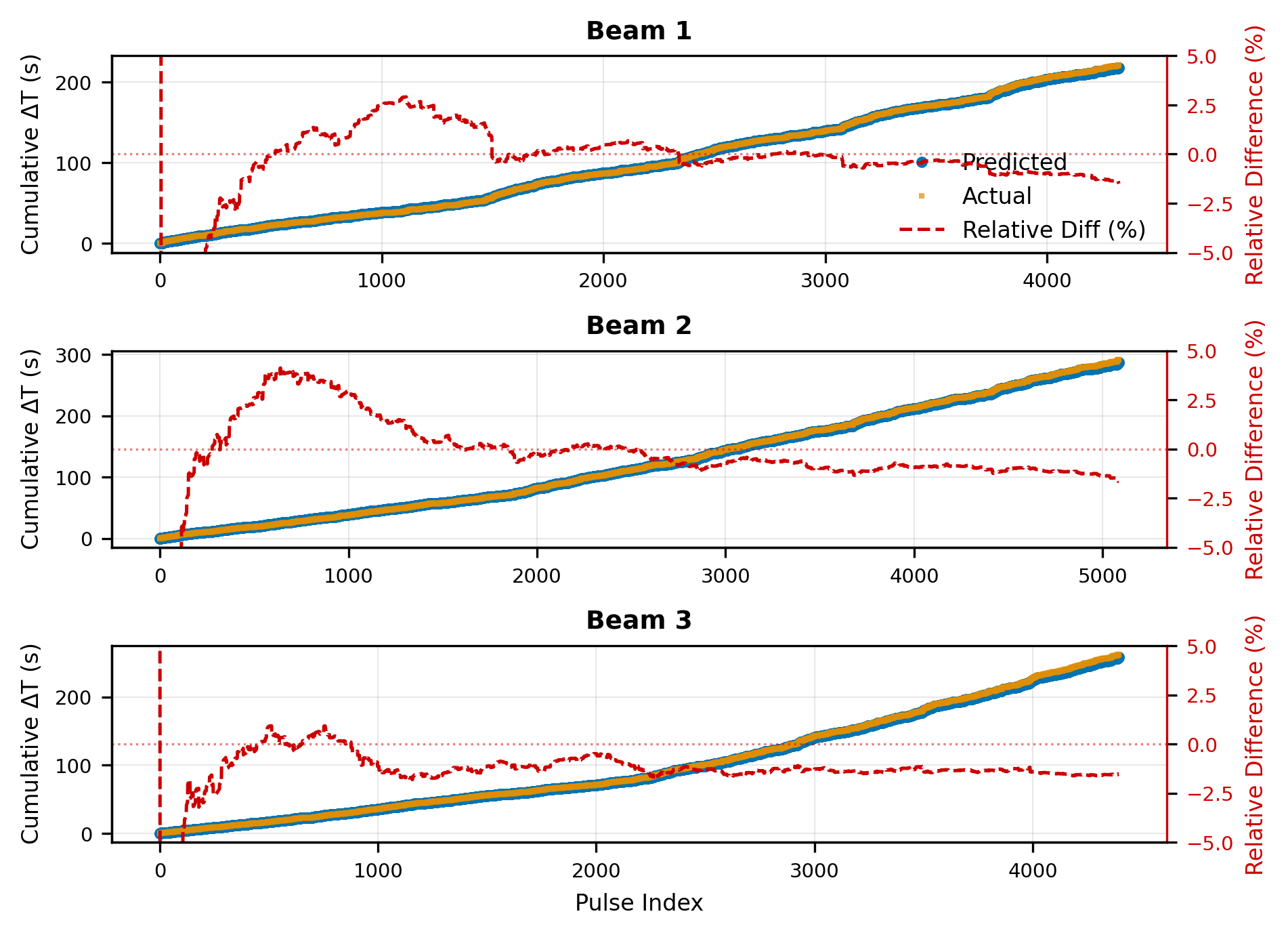


Figure S7. BDT predicted cumulative $\Delta T$ (blue dots) vs. actual log file values (orange dots) as a function of pulse index from the repainting clinical plan. The red dashed line shows the relative difference in percentage between the two.

# Declaration of Generative AI and AI-assisted technologies in the writing process

During the preparation of this work, the authors used Microsoft Copilot to review and enhance the clarity and grammar of the manuscript. After using this tool/service, the author(s) reviewed and edited the content as needed and take full responsibility for the content of the publication.

# References

[1] Henrotin S, Abs M, Forton E, Jongen Y, Kleeven W, Verbruggen P, et al. Commissioning and testing of the first IBA S2C2. Proc. 21st Int. Conf. on Cyclotrons and Their Applications (Cyclotrons-16), 2016, p. 178–80.

[2] Vilches-Freixas G, Unipan M, Rinaldi I, Martens J, Roijen E, Almeida IP, et al. Beam commissioning of the first compact proton therapy system with spot scanning and dynamic field collimation. Br J Radiol 2020;93:20190598.

[3] Volz L, Sheng Y, Durante M, Graeff C. Considerations for upright particle therapy patient positioning and associated image guidance. Front Oncol 2022; 12: 930850 2022.

[4] MEVION S250-FIT PROTON THERAPY SYSTEM n.d.

[5] Silvus A, Haefner J, Altman MB, Zhao T, Perkins S, Zhang T. Dosimetric evaluation of dose shaping by adaptive aperture and its impact on plan quality. Medical Dosimetry 2024;49:30–6.

[6] Engwall E, Mikhalev V, Sundström J, Marthin O, Wase V. Shoot-through layers in upright proton arcs unlock advantages in plan quality and range verification. Med Phys 2025;52:e18051.

[7] Janson M, Glimelius L, Fredriksson A, Traneus E, Engwall E. Treatment planning of scanned proton beams in RayStation. Medical Dosimetry 2024;49:2–12.

[8] Li H, Li Y, Zhang X, Li X, Liu W, Gillin MT, et al. Dynamically accumulated dose and 4D accumulated dose for moving tumors. Med Phys 2012;39:7359–67.

[9] Li Y, Kardar L, Li X, Li H, Cao W, Chang JY, et al. On the interplay effects with proton scanning beams in stage III lung cancer. Med Phys 2014;41:21721.

[10] Zhao L, Liu G, Zheng W, Shen J, Lee AK, Yan D, et al. Assessing the Interplay Effect Based on a Precise Machine-Specific Delivery Sequence and Time for Cyclotron Accelerator Proton Therapy System. Int J Radiat Oncol Biol Phys 2021;111:e529–e530.

[11] Rana S, Rosenfeld AB. Investigating volumetric repainting to mitigate interplay effect on 4D robustly optimized lung cancer plans in pencil beam scanning proton therapy. J Appl Clin Med Phys 2021;22:107–18.

[12] Mein S, Wuyckens S, Li X, Both S, Carabe A, Vera MC, et al. Particle arc therapy: Status and potential. Radiotherapy and Oncology 2024;199:110434.

[13] Meijers A, Daartz J, Knopf A-C, Van Heerden M, Bizzocchi N, Vazquez MV, et al. Possible association of dose rate and the development of late visual toxicity for patients with intracranial tumours treated with pencil beam scanned proton therapy. Radiation Oncology 2024;19:75.

[14] Zhao L, Liu G, Zheng W, Shen J, Lee A, Yan D, et al. Building a precise machine-specific time structure of the spot and energy delivery model for a cyclotron-based proton therapy system. Phys Med Biol 2022;67:01NT01.

[15] Zhao L, Liu G, Chen S, Shen J, Zheng W, Qin A, et al. Developing an accurate model of spot-scanning treatment delivery time and sequence for a compact superconducting synchrocyclotron proton therapy system. Radiation Oncology 2022;17:87.

[16] Liang X, Liu C, Furutani KM, Shen J, Bues M, Dougherty JM, et al. Investigation of beam delivery time for synchrotron-based proton pencil beam scanning system with novel scanning mode. Phys Med Biol 2022;67:175001.

[17] Burguete J, Garc\’\ia-Cardosa M, Antol\’\in E, Aguilar B, Azcona JD. Stochastic model for predicting the temporal structure of the plan delivery in a synchrotron-based pencil beam scanning proton therapy system. Radiation Physics and Chemistry 2025;226:112276.

[18] Wildman VL, Wynne J, Momin S, Kesarwala AH, Yang X. Recent Advances in Applying Machine Learning to Proton Radiotherapy. Biomed Phys Eng Express 2025.

[19] Missiaggia M, Pierobon E, La Tessa C, Cordoni FG. An exploratory study of machine learning techniques applied to therapeutic energies particle tracking in microdosimetry using the novel hybrid detector for microdosimetry (hdm). Phys Med Biol 2022;67:185002.

[20] Cordoni FG, Missiaggia M, Scifoni E, La Tessa C. An artificial intelligence-based model for cell killing prediction: development, validation and explainability analysis of the ANAKIN model. Phys Med Biol 2023;68:85017.

[21] Neishabouri A, Bauer J, Abdollahi A, Debus J, Mairani A. Real-time adaptive proton therapy: An AI-based spatio-temporal mono-energetic dose calculation model (CC-LSTM). Comput Biol Med 2025;188:109777.

[22] Padannayil NM, Sharma DS, Nangia S, Patro KC, Gaikwad U, Burela N. IMPT of head and neck cancer: unsupervised machine learning treatment planning strategy for reducing radiation dermatitis. Radiation Oncology 2023;18:11.

[23] Ho TK. Random decision forests. Proceedings of 3rd international conference on document analysis and recognition, vol. 1, 1995, p. 278–82.

[24] Ho TK. The random subspace method for constructing decision forests. IEEE Trans Pattern Anal Mach Intell 1998;20:832–44.

[25] Pedregosa F, Varoquaux G, Gramfort A, Michel V. and Thirion B, Grisel O, Blondel M, et al. Scikit-learn: Machine Learning in Python. Journal of Machine Learning Research 2011;12:2825–30.

[26] Lundberg SM, Lee S-I. A unified approach to interpreting model predictions. Adv Neural Inf Process Syst 2017;30.

[27] Lundberg SM, Erion G, Chen H, DeGrave A, Prutkin JM, Nair B, et al. From local explanations to global understanding with explainable AI for trees. Nat Mach Intell 2020;2:56–67.

[28] Cartechini G, Kneepkens E, Vilches-Freixas G, Lubken I, Velders M, Nijsten S, et al. In vivo and predictive interplay evaluation methodology for lung and esophageal cancer patients treated in free breathing with IMPT 2025.

[29] Friedman JH. Greedy function approximation: a gradient boosting machine. Ann Stat 2001:1189–232.

[30] Wuyckens S, Janssens G, Vera MC, Sundström J, Di Perri D, Sterpin E, et al. Proton arc therapy plan optimization with energy layer pre-selection driven by organ at risk sparing and delivery time. Phys Med Biol 2025;70:45003.

[31] Butkus M, Bastawros D, Yang Y, Cassetta R, Hytonen R, Kaderka R. Spot-optimization reduces beam delivery time in liver breath hold intensity modulated proton therapy. Phys Imaging Radiat Oncol 2025;34:100763.

[32] Santos A, Penfold S, Gorayski P, Le H. The role of hypofractionation in proton therapy. Cancers (Basel) 2022;14:2271.

[33] Liu S, Wu Y, Wooten HO, Green O, Archer B, Li H, et al. Methods to model and predict the ViewRay treatment deliveries to aid patient scheduling and treatment planning. J Appl Clin Med Phys 2016;17:50–62.
